# Intracellular sensing of viral genomes and viral evasion

**DOI:** 10.1038/s12276-019-0299-y

**Published:** 2019-12-11

**Authors:** Hyun-Cheol Lee, Kiramage Chathuranga, Jong-Soo Lee

**Affiliations:** 1 College of Veterinary Medicine, Chungnam National University, Daejeon, 34134 Korea; 2 Central Research Institute, Komipharm International Co., Ltd, Shiheung, 15094 Korea

**Keywords:** Immunology, Innate immunity

## Abstract

During viral infection, virus-derived cytosolic nucleic acids are recognized by host intracellular specific sensors. The efficacy of this recognition system is crucial for triggering innate host defenses, which then stimulate more specific adaptive immune responses against the virus. Recent studies show that signal transduction pathways activated by sensing proteins are positively or negatively regulated by many modulators to maintain host immune homeostasis. However, viruses have evolved several strategies to counteract/evade host immune reactions. These systems involve viral proteins that interact with host sensor proteins and prevent them from detecting the viral genome or from initiating immune signaling. In this review, we discuss key regulators of cytosolic sensor proteins and viral proteins based on experimental evidence.

## Viral infection: Evasion strategies subvert antiviral defenses

Viruses escape the body’s immune surveillance mechanisms by manipulating and subverting key intracellular sensors of viral RNA or DNA. In a review article, Jong-Soo Lee and colleagues from Chungnam National University in Daejeon, South Korea, discuss the strategies used by viral pathogens to avoid detection by immune receptors or to block activation of these receptors and the associated signaling molecules, thus preventing expression of antiviral genes. These strategies include modifying viral nucleic acids or making them inaccessible, and interference with sensor proteins, either though degradation, altered processing or relocalization within the cell. The authors summarize rapid advances in scientists’ understanding of sensor-mediated antiviral responses at the molecular level, and highlight how that knowledge could help guide the development of novel vaccines and antiviral agents.

## Introduction

Viral infection is a major threat to human and animal health worldwide. Acute and chronic infections cause many economic and social problems. Over the past few decades, the field of molecular cell biology has contributed to our knowledge of both viruses and the host innate immune reactions that they trigger. In particular, we now understand how host cells recognize invading viruses and how the antiviral signaling cascade is regulated.

Host innate immunity is the first line of defense against viral infection. Efficient and rapid detection of invading viruses, coupled with mechanisms that distinguish viral components from host components, is a critical factor. Upon viral infection, virus-derived pathogen-associated molecular patterns (PAMPs), such as viral capsid proteins, surface glycoproteins, and the viral genome, are recognized by host pattern recognition receptors (PRRs). There are several types of PRRs, which are identified according to cellular localization and ligand specificity; these include Toll-like receptors, C-type lectin receptors, retinoic acid-inducible gene-I (RIG-I)-like receptors (RLRs), nucleotide-binding oligomerization domain (NOD)-like receptors, and cytosolic DNA sensors such as cyclic GMP-AMP synthetase^1^. Sensing of viral PAMPs by PRRs triggers signaling cascades via adapter proteins such as mitochondrial antiviral signaling protein (MAVS) or stimulator of interferon genes (STING), ultimately leading to the production of host defense molecules such as type I and III interferons (IFNs), proinflammatory cytokines, and chemokines^2^. Secreted IFNs and cytokines enhance innate immune responses via autocrine and paracrine mechanisms and induce expression of interferon-stimulated genes (ISGs) that inhibit viral replication and spread^3^. Secreted cytokines and chemokines are also critical for inducing effective adaptive and memory immune responses.

Nonetheless, excessive production of IFNs and prolonged inflammatory responses triggered by uncontrolled PRR signaling can have deleterious effects on the host by promoting the development of autoimmune disorders, allergies, and other immunopathologies^4^. In contrast, weak or ineffective PRR signal transduction exacerbates the severity of viral disease. Therefore, PRR-mediated signal transduction must be tightly regulated (either positively or negatively) to maintain host immune homeostasis^5^.

In addition, viruses have evolved several strategies to avoid detection of host antiviral immune responses; these range from interruption of viral sensors to manipulation of molecules within signaling cascades^6^. For example, the viral genome harbors structures that mask specific molecular motifs recognized by cytosolic sensors. Some viral proteins inhibit host sensor molecules by cleaving or mediating degradation of signaling molecules or by interfering with post-translational modifications (PTMs) of sensors^6^. From the perspective of the virus, these actions during the early phase of invasion are critical for successful infection.

Here, we summarize recent evidence regarding interactions between key intracellular sensors, viral RNA/DNA, and molecules that regulate efficient IFN responses or maintenance of host immune homeostasis. Furthermore, we describe recent advances in our knowledge about viral evasion of host cytosolic sensors, focusing on interactions between cytosolic sensors and specific viral proteins.

## Host viral RNA sensors and viral evasion mechanisms

Upon viral infection, the viral genome is released into the cytoplasm to initiate viral protein biosynthesis. During this step, conserved molecular structures such as triphosphates and double-stranded (ds)RNA act as PAMPs that are recognized by sensors in the host cell cytosol (Table 1). The host innate immune system includes receptors, called PRRs, that distinguish the viral genome from the host genome. To achieve this, RLRs comprising RIG-I, melanoma differentiation-associated protein 5 (MDA5), laboratory of genetics and physiology 2 (LGP2), and other sensors such as NACHT, LRR, PYD domain-containing protein 3 (NLRP3), and nucleotide-binding oligomerization domain-containing protein 2, act as intracellular viral RNA sensors^7^. These proteins bind to viral RNA in the cell cytoplasm via RNA binding motifs, after which their signaling domain interacts with downstream adapter molecules, resulting in the activation of signaling cascades. The reactions are triggered as an immediate response to infection by RNA viruses and result in the production of type I IFNs, proinflammatory cytokines, and chemokines^2,8^. However, RNA viruses possess an arsenal of mechanisms to attenuate innate immune responses. Below, we describe the activation and regulation processes of major sensor molecules and mechanisms by which viruses evade them.

**Table 1:** Summary of RNA and DNA viruses and ligand recognition by PRRs

PRR	Agonist	Representative virus
RIG-I	5' ppp dsRNA short dsRNA 5' ppp ssRNA AU-rich 3' UTR RNase L cleavage products Circular viral RNA pU/UC HCV genomic RNA	SeV, NDV, RSV, MV, VSV, IAV, EBOV, JEV, HCV, WNV, DENV, Rotavirus, Vaccinia virus, Adenovirus, Rift Valley fever virus, Lassa virus, Nipha virus, Rabies virus, Influenza B virus
MDA5	Long dsRNA RNase L cleavage products AU-rich motifs	ECMV, MV, WNV, SeV, DENV, MHV, HCV, PIV5, EV, Murine norovirus-1, Rabies virus, Saffold virus, Rotavirus, Adenoviruses, Theiler’s virus
LGP2	dsRNA	ECMV, VSV, HCV, Poliovirus
cGAS	RNA:DNA intermediate dsDNA ssDNA Mitochondrial DNA	HSV-1, MHV68, Adenovirus
IFI16	dsDNA ssDNA	HSV-1, HCMV, KSHV, EBV
AIM2	dsDNA	MCMV, Vaccina virus

*dsRNA* double-stranded RNA, *ssRNA* single-stranded RNA, *UTR* untranslated region, *dsDNA* double-stranded DNA, *ssDNA* single-stranded DNA

## RIG-I

RIG-I, which belongs to the DExD/H box RNA helicase family, is an intracellular sensor of viral RNA. RIG-I recognizes 5′ tri- or di-phosphorylated dsRNA, the AU-rich 3′untranslated region (UTR), RNase L cleavage products, and circular viral RNA^9,10^. RIG-I detects the genomes of viruses such as vesicular stomatitis virus (VSV), influenza A virus (IAV), Sendai virus (SeV), Newcastle disease virus (NDV), respiratory syncytial virus (RSV), hepatitis C virus (HCV), and Japanese encephalitis virus (JEV)^10–12^. In addition, some DNA viruses such as vaccinia virus and Herpes simplex virus (HSV)^9^ and bacteria such as *Listeria monocytogenes* generate RNA that is then targeted by RIG-I^13^. Structurally, RIG-I comprises two N-terminal caspase activation and recruitment domains (CARDs), two helicase domains (Hel-1 and Hel-2), and a C-terminal repressor domain (RD)^14^. In the resting state, RIG-I is autoinhibited by its own RD. In response to virus invasion, RIG-I recognizes viral RNA via its two components: the RD and helicase domain. The RD facilitates viral RNA recognition through its strong affinity for the 5′ end triphosphate, and the positively charged pocket structure of the RD interacts with the 5′ end of viral RNA^15,16^. The helicase domain binds to dsRNA and mediates a conformational change that allows ATP binding to activate RIG-I^15,16^. This conformational change opens up the CARDs, which are essential for downstream signaling^14,17^. During this step, RIG-I is activated or inactivated by several regulators and/or PTMs (see below). Open CARDs interact with the CARD MAVS to activate downstream signaling cascades^18^. In addition, adapters such as TNF receptor associated factor (TRAF) 3 or TRAF6, serine/threonine-protein kinases, TANK-binding kinase (TBK1), and IκB kinase (IKK) are activated^9,10^. Consequently, transcription factors such as IRF3, IRF7, and nuclear factor kappa-light-chain-enhancer of activated B cells (NF-κB) trigger production of type I IFNs and induce expression of antiviral molecules^9,10^.

**Fig. 1:**
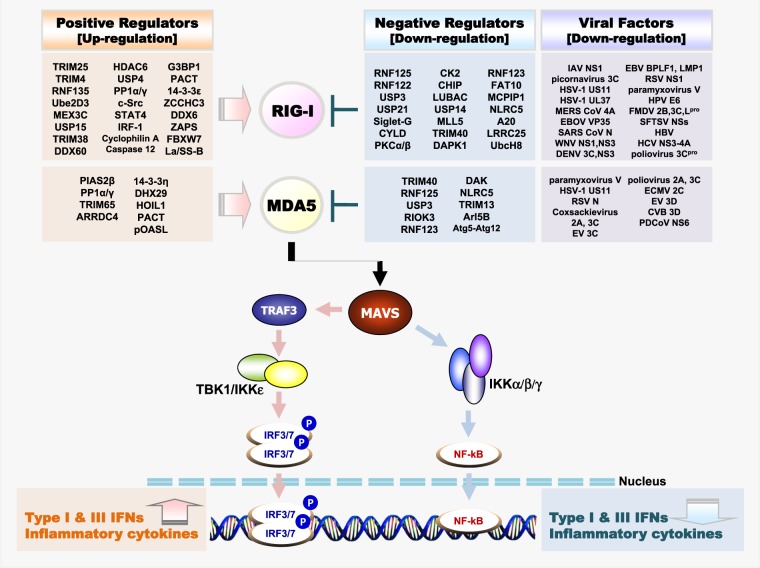
Regulators and interacting viral proteins of the RLR–MAVS antiviral signaling pathway. Schematic presentation of positive and negative regulators of RLRs (Top) and melanoma differentiation-associated protein-5 (MDA5) (Bottom) through PTMs or non-PTMs and immune invasion viral proteins interacting with RIG-I (Top) and MDA5 (Bottom). The RLR-MAVS pathway includes the key cytosolic sensors RIG-I and MDA5, which detect viral RNA. These sensors subsequently interact with the central antiviral signaling protein MAVS, which in turn activates the transcription factors NF-κB and IRF3/IRF7 via the cytosolic kinases IKK and TBK1/IKKε, respectively. Activated transcription factors NF-κB, IRF7 and IRF3 translocate to the nucleus and induce transcription of type I IFN and pro-inflammatory genes

RIG-I is essential for innate antiviral immunity; however, it is modulated by several regulatory molecules to protect against viral spread or the maintenance of host immune homeostasis (Fig. 1). First, RIG-I activation or inactivation is regulated by PTMs such as ubiquitination, phosphorylation, and acetylation^5^. During activation, RIG-I undergoes K63-linked ubiquitination by RING finger protein 135 (RNF135/Riplet), tripartite motif-containing protein (TRIM4), and TRIM25^19–23^. Importantly, K63-linked ubiquitination of the CARD at K172 is mediated by TRIM25, which induces RIG-I oligomerization^22^. Caspase 12 promotes K63-mediated ubiquitination of RIG-I via TRIM25 to promote RIG-mediated signaling, whereas linear ubiquitin chain assembly complex (LUBAC) negatively regulates TRIM25 via K48-linked ubiquitination to trigger proteasomal degradation^24,25^.

Conversely, ubiquitin carboxyl-terminal hydrolase (USP) 15 mediates deubiquitination of K48-linked ubiquitination of TRIM25^26^. In addition, mex-3 RNA-binding family member C (MEX3C) mediates K63-linked ubiquitination of RIG-I to promote the formation of stress granules, which generate a platform complex for viral sensing and signaling^27^. In contrast to activation by K63-linked ubiquitination, removal of K63-linked polyubiquitin by the deubiquitinating enzyme CYLD negatively regulates RIG-I activity^28^. Two other deubiquitinases, USP3 and USP21, also negatively control RIG-I activity in the same way^29,30^. K63-linked ubiquitination of RIG-I by TRIM25, MEX3C, and TRIM4 and deubiquitination of RIG-I by CYLD, USP3, and USP21 occur in the CARDs, whereas K63-linked ubiquitination by the RNF135/Riplet occurs in the RD (K788), which has a positive effect on TRIM25-mediated K63-linked ubiquitination in the CARDs^19,20,23,28–30^. In contrast, K48-linked ubiquitination serves as a signal for proteasomal degradation of RIG-I. RNF122 and RNF125 mediate K48-linked ubiquitination, which inhibits RIG-I-mediated antiviral innate immune responses^31,32^. Sialic acid binding Ig-like lectin G (Siglec-G) recruits the E3 ligase c-cbl to RIG-I, resulting in degradation via K48-linked ubiquitination^33^. The deubiquitinase USP4 also serves as a negative regulator of ubiquitination^34^.

Phosphorylation and acetylation are also important PTMs involved in RIG-I regulation. In resting cells, for example, RIG-I is autoinhibited by phosphorylation and acetylation. Phosphorylation on the CARDs of RIG-I is maintained by protein kinase C (PKC) α/β and removed by protein phosphatase 1 (PP1) α/γ, which induces further K63-linked ubiquitination at this domain^35,36^. Casein kinase II (CK2) phosphorylates the RD of RIG-I, and removal of this phosphorylation allows for K63-linked ubiquitination via RNF135^37^. When RIG-I is inactivate, the RD domain is acetylated. In the presence of RNA ligands, histone deacetylase (HDAC) 6 deacetylates the RD and allows RIG-I to bind to viral RNA to promote oligomerization^38,39^. However, some regulators involved in RIG-I function do not act via PTMs. As a positive regulator, the shorter isoform of PARP-13 (ZAPS) associates with RIG-I and promotes its oligomerization, whereas IRF1 functions to increase expression of RIG-I^40,41^. The mitochondrial targeting chaperone protein 14-3-3ε interacts with RIG-I, thereby translocating it to the translocon^42^. As a negative regulator, RNF123 blocks RIG-I and inhibits signaling of MAVS without its E3 ligase function^43^. Caspase-8 is recruited to RIG-I upon viral infection, whereupon it cleaves the RIG-I signaling enhancer receptor-interacting protein (RIP) 1^44^. Other molecules that negatively regulate RIG-I are listed in Table 2.

**Table 2:** Regulators for RNA and DNA virus PRRs

PRR	Classification		Regulator	Function	Ref.
**RIG-I**	**PTMs**	**Positive**	TRIM4	K63 ubiquitination	^21^
			TRIM25	K63 ubiquitination	^20^
			RNF135/Riplet/REUL	K63 ubiquitination	^17–19^
			Ube2D3/Ube2N	K63 ubiquitination	^126^
			Cyclophilin A	TRIM25-mediated ubiquitination	^127^
			MEX3C	K63 ubiquitination Antiviral stress granule	^25^
			USP15	TRIM25 deubiquitylation	^24^
			HDAC6	Deacetylation	^36,37^
			Caspase 12	TRIM25-mediated ubiquitination	^23^
			USP4	Deubiquitination	^32^
			PP1α/γ	Dephosphorylation	^34^
			WHIP-TRIM14-PPP6C	Dephosphorylation	^128^
			c-Src	TRIM25 phophorylation	^129^
			TRIM38	SUMOylation	^130^
			STAT4	Blocking CHIP	^131^
		**Negative**	RNF122	K48 ubiquitination	^30^
			USP3	Deubiquitination	^27^
			USP21	Deubiquitination	^28^
			Siglec-G/c-Cbl	K48 ubiquitination	^31^
			PKCα/β	Phosphorylation	^33^
			CK2	Phosphorylation	^35^
			CYLD	Deubiquitination	^26^
			CHIP	K48 ubiquitination	^131^
			RNF125	Proteasomal degradation	^29^
			LUBAC	TRIM25 degradation	^22^
			USP14	Deubiquitination	^132^
			MLL5	CHIP mediated ubiquitination	^133^
			TRIM40	K27, K48 ubiquitination	^134^
			DAPK1	Phosphorylation	^135^
			SENP2	DeSUMOylation	^135^
	**Non-PTMs**	**Positive**	G3BP1	Antiviral stress granule	^136^
			PACT	Physical interaction	^137^
			14-3-3ε	Translocation	^40^
			ZCCHC3	Physical interaction TRIM25-mediated ubiquitination	^138^
			DDX6	Physical interaction Antiviral stress granule	^139^
			La/SS-B	Physical interaction	^140^
			FBXW7	Stabilization	^141^
			ZAPS	Physical interaction	^39^
			IRF1	Expression level enhancing	^38^
			DDX60	Physical interaction	^62^
		**Negative**	RNF123	Physical interaction	^41^
			KHSRP	Physical interaction	^142^
			MCPIP1	Expression level reducing	^143^
			FAT10	Antiviral stress granule	^144^
			RIP-Caspase8	Facilitating RIG-I complex by RIP1/Cleavage of RIP1 by Caspase8	^42^
			NLRC5	Physical interaction	^66^
			SEC14L1	Physical interaction	^145^
			Atg5-Atg12	Physical interaction	^65^
			A20	Physical interaction	^146^
			LRRC25	Autophagic degradation	^147^
			UbcH8	ISG15 conjugation	^148^
**MDA5**	**PTMs**	**Positive**	PIAS2β	SUMOylation	^59^
			PP1α/γ	Dephosphorylation	^34^
			TRIM65	K63 ubiquitination	^61^
			ARRDC4	TRIM65 mediated ubiquitination	^149^
		**Negative**	TRIM40	K27, K48 ubiquitination	^139^
			RNF125	Proteasomal degradation	^29^
			USP3	Deubiquitination	^27^
			RIOK3	Phosphorylation	^63^
	**Non-PTMs**	**Positive**	14-3-3η	Oligomerization Intracellular redistribution	^150^
			DHX29	Aggregation with MDA5	^151^
			HOIL1	Association	^152^
			PACT	MDA5 Oligomerization	^75^
			pOASL	Physical interaction	^153^
		**Negative**	DAK	Physical interaction	^154^
			NLRC5	Physical interaction	^66^
			TRIM13	Physical interaction	^60^
			Atg5-Atg12	Physical interaction	^65^
			Arl5B	Physical interaction	^155^
			RNF123	Physical interaction	^41^
**LGP2**	**Non-PTMs**	**Positive**	PACT	Physical interaction	^75^
			PUM1	Physical interaction	^74^
**cGAS**	**PTMs**	**Positive**	TRIM56	Monoubiquitination	^87^
			TRIM14-USP14	Deubiquitination	^84^
			RNF185	K27 ubiquitination	^85^
			CCP5	Deglutamylation	^90^
			CCP6	Deglutamylation	^90^
			TRIM38	SUMOylation	^88^
			SENP7	DeSUMOylation	^89^
			RINCK (TRIM41)	Monoubiquitination	^86^
			TTLL4	Monoglutamylation	^99^
			TTLL6	Polyglutamylation	^99^
			SENP2	DeSUMOylation	^88^
			Akt	Phosphorylation	^91^
			HDAC3	Deacetylation	^92^
	**Non-PTMs**	**Positive**	PI(4,5)P2	Physical interaction plasma membrane localization	^92^
			G3BP1	Changing the structure Oligomerization	^94^
			ZCCHC3	Physical interaction	^95^
		**Negative**	OASL (Human)	Enzyme activity inhibition	^97^
			Oasl2 (Mouse)	Enzyme activity inhibition	^97^
			Caspase-1	Cleavage	^98^
			Caspase-4/5/11	Cleavage	^98^
			Beclin-1	Physical interaction	^96^
**IFI16**	**PTMs**	**Positive**	P300	Acetylation	^108^
	**Non-PTMs**	**Positive**	cGAS	Physical interaction	^109,116^
			ASC, procaspase-1	Physical interaction	^106,107^
			BRCA1	translocation	^110^
**AIM2**	**Non-PTMs**	**Positive**	HMGB1	Physical interaction	^119^
		**Negative**	TRIM11 and p62	Autophagic degradation	^120^

RIG-I is tightly controlled by a wide range of regulatory factors. For structural activation and initiation of innate immune responses, RIG-I is involved in many specific modification mechanisms with other regulatory factors. Since the discovery that TRIM25 activates RIG-I via K63 ubiquitination, a burgeoning number of other factors regulating RIG-1 activity, such as PTMs, oligomerization, RNA recognition, relocalization, and stabilization, have been identified. Moreover, beyond these processes directly affecting RIG-1 activity, some of the factors that regulate RIG-1 are controlled by other factors. Taken together, these findings highlight the complexity and delicate control of RIG-I-mediated innate immune signaling.

## Viral evasion of RIG-I-mediated responses

In the early stage of viral infection, avoidance of innate immunity, including the interferon response, is important for successful viral infection. Because RIG-I is a key viral RNA sensor that initiates rapidly innate immune responses, it is targeted by diverse viral proteins.

Many viruses possess proteins that interfere directly with RIG-I. For example, HSV-1 tegument protein US11 interacts with RIG-I to block formation of the RIG-I-MAVS complex; porcine deltacoronavirus (PDCoV) accessory protein NS6 interferes with RIG-I binding to dsRNA^45,46^. The 3C protease of Enterovirus (EV) 71, Poliovirus, Echovirus, Rhinovirus type 16, Rhinovirus type 1A, and Encephalomyocarditis virus (EMCV) cleaves and inactivates RIG-I^47^. Latent membrane protein (LMP) 1 of Epstein–Barr virus (EBV) mediates proteasomal degradation of RIG-I, and the nonstructural (NS) protein of severe fever with thrombocytopenia syndrome virus (SFTSV) hijacks RIG-I and its signaling proteins in the cytoplasmic structure^48,49^. In addition, evidence suggests that human respiratory syncytial virus (HRSV) N and P proteins colocalize with RIG-I and that the influenza virus NS1 protein interacts with RIG-I directly^50,51^. Interaction between RIG-I and these viral proteins mainly leads to direct functional impairment of RIG-I. The processes used by these viral proteins include cleavage, degradation, suspension, and inhibition of RIG-I. Specific viral proteins also interfere with RIG-I activation. A number of viral proteins target TRIM25-mediated K63-linked ubiquitination of RIG-I. The NS1 protein of IAV targets TRIM25 to block K63-linked ubiquitination^52^, whereas Paramyxovirus V proteins, HRSV NS1, and the severe acute respiratory syndrome coronavirus (SARS-CoV) nucleocapsid protein target TRIM25 to inhibit activation of RIG-I^53–55^. Moreover, the human papillomavirus (HPV) E6 protein increases the activity of USP15 to promote proteasomal degradation of TRIM25, and Herpesvirus mediates autoubiquitination of TRIM25 to prevent K63-linked ubiquitination of RIG-I^56,57^. Furthermore, West Nile virus (WNV) NS1 interferes with the innate immune response by mediating proteasomal degradation of RIG-I and inhibiting K63-linked ubiquitination^58^. In addition, some viral proteins dephosphorylate RIG-I and inhibit its signaling, and measles virus (MV) activates the C-type lectin DC-SIGN and blocks PP1 activity to attenuate RIG-I in dendritic cells^59^. The viral proteins that interact with or affect RIG-I are listed in Table 3.

**Table 3:** Viral evasion mechanism for RNA and DNA virus PRRs

PRR	Virus	Virulence factor	Function	Ref.
RIG-I	IAV	NS1	TRIM25 inhibition	^50^
	Picornavirus Poliovirus, Rhinoviruses, Echovirus, EMCV	3Cpro	Cleavage	^45^
	CVB	3Cpro	Cleavage	^69^
	SFTSV	NSs	Physical interaction cytoplasmic structure	^46^
	MERS-CoV	4A	PACT suppression	^156^
	EBOV	VP35	Physical interaction	^157^
	Marburg Virus			
	SARS-CoV	N	TRIM25 inhibition	^51^
	EBV	BPLF1	TRIM25 autoubiquitination RIG-I signalosome inactivation	^158^
		LMP1	Proteasomal degradation	^47^
	RSV	NS1	TRIM25 inhibition	^52^
	Paramyxovirus	V	Physical interaction TRIM25 inhibition	^53^
	PDCoV	NS6	Physical interaction	^44^
	HPV	E6	USP15 activation	^54^
	Toscana virus	NSs	Proteasomal degradation	^159^
	FMDV	Lpro	Cleavage	^160^
		3A	Physical interaction	^161^
		2B	Expression level decreasing	^162^
	HBV	–	miR146a inducing	^163^
	HCV	NS3-4A	Cleavage of Riplet	^164^
	HSV	US11	Physical interaction	^43^
		UL37	Deamidation	^165^
	DENV	sfRNA	TRIM25 inhibition	^166^
		NS3	Translocation (14-3-3ε)	^167^
	WNV	NS3	Translocation (14-3-3ε)	^167^
		NS1	Proteasomal degradation	^56^
MDA5	Poliovirus	2Apro 3Cpro	Cleavage	^69^
	CVB	2Apro	Cleavage	^69^
	Paramyxovirus	V	Physical interaction	^67^
	HSV	US11	Physical interaction	^43^
	HRSV	N	Physical interaction Inclusion body formation	^48^
	CVA	3C	Physical interaction	^168^
	EV			
	PDCoV	NS6	Physical interaction	^44^
	ECMV	2C	Physical interaction	^169^
	EV71 CVB	3Dpol	Physical interaction	^170^
LGP2	FMDV	Lpro	Cleavage	^80^
		2B	Physical interaction	^80^
	Paramyxovirus	V Protein	Suppress interaction with MDA5	^76^
cGAS	KSHV	ORF52 (KicGAS)	Disrupts cGAS binding to DNA	^103^
		LANA	Physical interaction	^103^
	ZIKV	NS1	Cleave K11 polyubiquitin chains from caspase-1	^98^
	HSV	UL37	Deamidation of cGAS	^99^
		VP22	Enzyme activity inhibition	^102^
	HCMV	UL31	Disassociation DNA from cGAS	^102^
		pUL83	Direct binding Interrupts cGAS STING binding	^101^
	DENV	NS2B	Degradation	^104^
	HIV-1	Capsid	Sensing inhibition	^171^
IFI16	KSHV	Lytic proteins	Ubiquitination and proteosomal degradation	^112^
	HSV	ICP0, ICP8	Proteasomal degradation	^111^
	HCMV	pUL97	Phosphorylation Mis-localization	^172^
		Vps4, TGN46	Trafficking into multivesicular bodies	^115^
		pUL83	Direct binding, block oligomerization Physical interaction	^113,114^
AIM2	HCMV	pUL83	Physical interaction	^121^
	HSV	VP22	Block AIM2 oligomerization	^122^

Taken together, results to date show that viruses express specific proteins that interfere with RIG-I function via diverse mechanisms and are essential for viral pathogenesis.

## MDA5 and viral evasion

MDA5 is a major intracellular sensor that recognizes viral dsRNA, including the genomes of EMCV, Poliovirus, Coxsackievirus, Rotavirus, Dengue virus (DENV), WNV, and murine hepatitis virus^10,11^ (Table 3). MDA5 recognizes long dsRNA, AU-rich motifs, and RNase L cleavage products^10,11,60^. MDA5 activation is similar to that of RIG-I; however, the MDA5 RD binds to the RNA backbone and not to the 5′end. This difference allows the HEL2 loop of MDA5 insert to the major groove of viral RNA, which is not limited to the RNA end^16^. This interaction triggers the two CARDs to form a tetrameric structure that transduces a signal to the adapter molecule MAVS, which is shared with the RIG-I mediated pathway^16,61^.

Several regulators and PTMs also regulate MDA5 activation and inactivation (Fig. 1). MDA5 activation involves dephosphorylation of S88 on the MDA5 CARD by PP1α/γ^36^. MDA5 also undergoes SUMOylation by PIAS2β, which promotes interferon signaling^62^, and TRIM38 acts as a SUMO E3 ligase to mediate downstream signaling via SUMOylation of K43/K865 of MDA5^63^. K63-linked ubiquitination at the CARDs is also a critical mechanism underlying MDA5 activation. In addition, TRIM65 mediates K63-linked ubiquitination of K743 in the CTD of MDA5 to induce MDA5 oligomerization and activation^64^, and DEXD/H box helicase DDX60 also acts as a positive regulator by binding to MDA5^65^. In contrast, right open reading kinase 3 (RIOK) phosphorylates the RD of MDA5 at S828 to inhibit filament formation^66^. Notably, the E3 ubiquitin ligase RNF125 negatively regulates MDA5 via its ligase function, whereas RNF123 performs the same role independently of its ligase function^31,43^. USP3 and USP21 inhibit MDA5 function via deubiquitination^29,30^, and proteins such as dihydroxyacetone kinase (DAK), Atg5-Atg-12, NLRC5, and TRIM13 interact with and inhibit MDA5 (Table 2)^63,67–69^.

MDA5 is also subject to several types of activation that resemble those that activate RIG-I. Indeed, PP1α/γ, USP3, RNF123, and TRIM40 target RIG-I and MDA5 simultaneously, likely because the domain structures of these two proteins are similar. Upon activation, these two proteins transmit signals through their CARDs to MAVS and share subsequent pathways involved in cytokine secretion. Hence, these two proteins are commonly involved in virus recognition and play a complementary role in the initiation of innate immunity. Regardless, further studies on the molecular mechanisms that regulate MDA5 activation and inactivation are necessary.

Different viral proteins have been reported to inhibit MDA5. The V proteins of paramyxoviruses limit the induction of IFN-β by interfering with MDA5 but not RIG-I^70^. In addition, the V protein of Parainfluenza virus (PIV) 5, Mumps virus, MV, Menangle virus, Hendra virus, Nipah virus, Maquera virus, SeV, and Salem virus binds to MDA5^70^. In particular, structural studies have demonstrated that the V protein of PIV5 recognizes a structural motif within MDA5, thereby disrupting its ATP-hydrolysis function as well as filament formation^71^. The helicase C domain of MDA5 is sufficient for association with V proteins from PIV2, PIV5, MV, Mumps virus, Hendra virus, and Nipah virus. In addition, human herpesvirus tegument protein US11 and the HRSV N protein antagonize innate immune responses initiated by MDA5^45,50^. The 2A protease of Coxsackievirus B (CVB) 3 and Poliovirus mediates degradation of MDA5 in a proteasome- and caspase-dependent manner, whereas EV 71 2A cleaves MDA5 to inactivate it^72^.

## LGP2 and viral evasion

LGP2 is an RLR that lacks N-terminal CARDs, and thus LGP2 cannot transmit signals to MAVS; however, it can bind to viral RNA and modulate the activities of RIG-I and MDA5^11^. Overall, the exact role of LGP2 in innate immunity is still unclear, though based on previous studies, LGP2 is a negative regulator of RLRs^73,74^ and acts synergistically with MDA5^63,64^. Although recent evidence shows that LGP2 strengthens MDA5-mediated innate immune responses against HCV infection^75^, other studies suggest that LGP2 acts as a negative regulator by interacting with TRAF family proteins and interfering with their ubiquitin ligase activity^76^.

Because LGP2 lacks CARDs and signaling activity, few studies have examined how it is regulated. Nonetheless, research suggests that pumilio protein 1 (PUM1) regulates expression of innate immune genes by acting as a biphasic negative regulator of LGP2^77^. In addition, PACT amplifies innate immune responses when expressed together with both LGP2 and MDA5^78^.

Although the role of LGP2 is not clear, interactions between viruses and LGP2 have been reported. For example, the Paramyxovirus V protein interacts with LGP2 and interferes with its ability to coactivate MDA5^79^. Foot and mouth disease virus (FMDV) 2B also directly interacts with LGP2, and expression of LGP is decreased by the C-terminal region of 2B^80^. Furthermore, FMDV leader protease (Lpro) directly interacts with and cleaves LGP2^80^; this event is thought to affect the function of LGP2 that regulates MDA5, which is responsible for FMDV genome recognition. Further research will be required to address the functions or molecular mechanisms of LGP2 and how its activity is affected by viral proteins.

## Host viral DNA sensors and viral evasion mechanisms

Upon DNA virus invasion, viral DNA is released into the host cell cytoplasm, and viral protein synthesis begins. Because the DNA of eukaryotic cells is located in the nucleus or mitochondrion, the presence of viral DNA in the cytoplasm acts as a PAMP, which is detected by several intracellular sensor molecules^81^.

Based on recent reports, cytoplasmic viral DNA is recognized by cyclic GMP-AMP synthase (cGAS), interferon gamma inducible protein 16 (IFI16), interferon-inducible protein (AIM2), DDX41, and RNA PolIII, among others. Similar to detection of viral RNA, immediate detection of viral DNA triggers host innate immune responses and enhances expression of antiviral-related cytokines^74,81,82^. cGAS and IFI16 transmit signals to the endoplasmic reticulum (ER) adapter protein STING, whereas AIM2 and IFI16 mediate activation of the inflammasome^82,83^. Ultimately, these reactions activate type I IFN signaling and antiviral responses, similar to those observed in RLR signaling. The activities of DNA-sensing factors are also modulated by a number of positive and negative regulators. However, DNA viruses have evolved numerous and elaborate strategies to counteract viral DNA sensing by host sensor molecules. Below, we summarize the regulation of major sensor factors and viral evasion mechanisms.

## cGAS and viral evasion

cGAS is a well-known cytosolic DNA sensor essential for early innate immune responses to DNA viruses. In the cytoplasm, cGAS detects self and nonself DNA and induces production of type I IFNs and proinflammatory cytokines. Ligands for cGAS are present in the genomes of HSV-1, Kaposi’s sarcoma-associated herpesvirus (KSHV), Vaccinia virus, murine gammaherpesvirus 68 (MHV68), Adenovirus, and Hepatitis B virus (HBV)^18,74,82^. cGAS binds to the sugar-phosphate backbone of dsDNA without sequence specificity. This interaction is mediated by positively charged DNA-binding sites of cGAS and induces a conformational change in cGAS, opening activation sites for cGAMP synthesis^18,82^. After recognition of viral DNA, cGAS generates 2′,3′-cGAMP, along with ATP and GTP, all of which play roles as second messengers to activate STING^84,85^, which undergoes conformational changes and is translocated from the ER to ER-Golgi intermediate compartments^86^. TBK1 is also activated, resulting in phosphorylation of transcription factors that potentiate cytokine-mediated antiviral responses^82^.

**Fig. 2:**
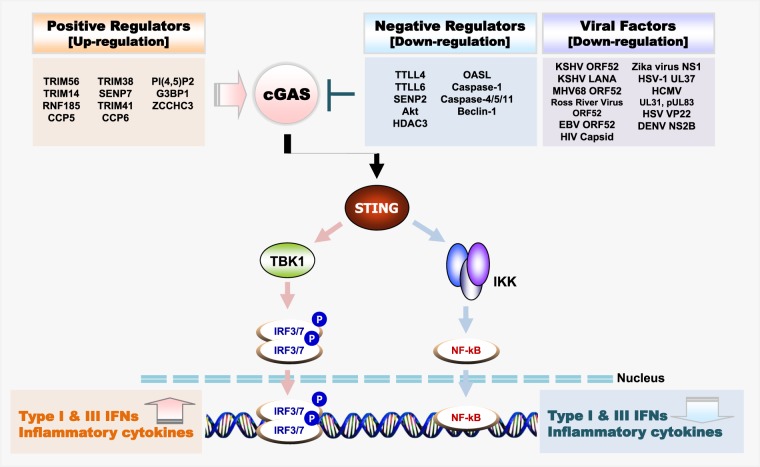
Regulators and interacting viral proteins of the cGAS–STING antiviral signaling pathway. Schematic presentation of positive and negative regulators of cGAS through PTMs or non-PTMs and immune invasion viral proteins interacting with cGAS. cGAS induces signaling through the adapter protein STING, resulting in dimerization of STING and activation of the transcription factors NF-κB and IRF3/IRF7 via cytosolic kinases IKK and TBK1, respectively. Activated transcription factors NF-κB, IRF7, and IRF3 translocate to the nucleus and induce transcription of type I IFN and pro-inflammatory genes

cGAS is also controlled by PTMs, such as phosphorylation, ubiquitination, SUMOylation, acetylation, and glutamylation^5^ (Fig. 2). Polyubiquitination of cGAS is mediated by E3 ligase, TRIM14, RINCK/TRIM41, and TRIM56^87–90^, whereas ER-resident RNF185 catalyzes K27-linked ubiquitination in response to HSV-1 infection. Importantly, RNF185-mediated ubiquitination at K173 and K384, two major ubiquitination sites, activates cGAS, thus generating more cGAMP^88^. RINCK/TRIM41 and TRIM56 mediate cGAS monoubiquitination and promote innate immune responses to DNA viruses; cGAS K335 is also monoubiquitinated by TRIM56 and E1 and UbcH5 E2 enzymes^89,90^.

K48-linked ubiquitination of cGAS is a recognition signal that triggers selective autophagic degradation, whereby TRIM14 recruits USP14 to cleave K48-linked ubiquitination and stabilize cGAS^87^. SUMOylation also plays an important role in regulating cGAS. TRIM38 mediates SUMOylation of cGAS to inhibit its degradation, whereas sentrin-specific protease (SENP) 2 induces SUMOylation and ultimately degradation of cGAS^91,92^. SENP7 targets cGAS for deSUMOylation, thereby stabilizing it and protecting it from degradation^92^. cGAS is also regulated by glutamylation, phosphorylation, and acetylation. Tubulin tyrosine ligase-like (TTLLs) glutamylases 4 and 6 target E302 for monoglutamylation and E272 for polyglutamylation, respectively, whereas cytosolic carboxypeptidases (CCP) 5 and 6 antagonize TTLLs^93^. Phosphorylation of cGAS at S305 and S291, as catalyzed by protein kinase B (PKB/Akt), strongly suppresses cGAS^94^. In addition, acetylation inhibits cGAS-mediated production of interferon^95^. There are also other mechanisms of cGAS regulation that do not rely on PTMs. As positive regulators, manganese and the Ras-GAP SH3 domain-binding protein (G3BP1) target cGAS to promote its DNA-binding activity^96,97^; PI(4,5)P2 localizes cGAS to the plasma membrane^95^. The CCHC-type zinc-finger (ZF) protein ZCCHC3 acts with cGAS as a cosensor to enable recognition of dsDNA^98^. In contrast, inflammasome activation triggers caspase-mediated cleavage of cGAS. Oligoadenylate-synthetase-family (OASL) protein downregulates cGAS enzyme activity, and becline 1 targets cGAS to suppress cGAMP synthesis^99,100^. Recent studies have also reported glutamylation and monoubiquitination as novel PTMs of cGAS, and two novel carboxypeptidases and glutamylases regulate cGAS via differential glutamylation^93^. TRIM56 monoubiquitinates cGAS and affects antiviral signaling by promoting a marked increase in cGAS dimerization and DNA-binding activity, which eventually increases cGAMP production^90^.

To antagonize host innate immune activation, Zika virus (ZIKV) NS1 stabilizes caspase 1 and protects it from proteasomal degradation^101^. K11-linked ubiquitin chains of caspase 1 at K134 are cleaved by USP8, which is recruited by NS1. Thus, ZIKV triggers degradation of cGAS by caspase 1, thereby blocking antiviral innate immunity^101^. HSV-1 also evades cGAS-mediated innate immune responses through two viral proteins: the UL37 tegument protein deamidates cGAS, a process that determines species-specific inactivation of HSV-1^102^; and VP22 of HSV-1 interacts with cGAS to inhibit its enzymatic activity^103^.

To evade cGAS-mediated innate immune responses, UL31 of human cytomegalovirus (HCMV) interacts with cGAS to inhibit cGAMP synthesis; this is achieved by preventing cGAS from binding to DNA, whereas pp65 of HCMV inhibits cGAS activity^104,105^.

ORF52 and LANA of KSHV also inhibit cGAS activity. LANA of KSHV interacts with cGAS directly to inhibit cGAS-mediated pathways, whereas ORF52 of KSHV prevents cGAS from sensing DNA by inhibiting its enzymatic activity^106^. In addition, cGAS detects mitochondrial DNA released during DENV infection; however, the NS2B of DENV mediates lysosomal degradation of cGAS^107^.

The mechanisms by which virus proteins interfere with cGAS involve inhibition of DNA-binding activity and enzymatic activity or degradation. In particular, HSV-1 and HCMV express multiple proteins that interfere with cGAS-triggered innate immunity^102–105^. It is thought that many viruses have evolved diverse mechanisms that hinder cGAS function because cGAS is an intracellular sensor that is critical for detecting viral DNA. cGAS is inhibited not only by DNA viruses but also by RNA viruses such as ZIKV and DENV^101,107^. Moreover, DNA as a byproduct of RNA viral infection is recognized by cGAS, and viruses possess a mechanism to avoid this type of recognition. Taken together, these findings suggest that viral pathogenesis involving host immunity is more complex and sophisticated than previously thought, indicating that more research is needed in this area.

## IFI16 and viral evasion

IFI16 is a nuclear protein located predominantly in the nucleus; however, it shuttles between the nucleus and cytoplasm to sense viral DNA derived from Herpesvirus, human immunodeficiency virus (HIV), and bacteria such as listeria monocytogenes^74,81^. IFI16 contains an N-terminal pyrin domain (PYD) and two C-terminal HIN domains: it recognizes viral DNA via the HIN domain and then interacts with cGAS to promote cGAMP production and plays a vital role in cGAMP-mediated signaling, which activates TBK1 within the STING complex^5,108^. IFI16 is also able to detect viral DNA in the nucleus, activating ASC, an adapter molecule for the inflammasome and leading to production of IL-1β and IL-18^109,110^.

As IFI16 recognizes the Herpesvirus genome, it has been the subject of intense study. Upon Herpesvirus infection, IFI16 is acetylated by P300 in the nucleus and activates STING after its translocation to the cytoplasm^111^. cGAS also stabilizes IFI16 to promote innate immune signaling during HSV infection, though cGAS generates less cGAMP^112^. BRCA1 forms a complex with IFI16 in the nucleus that is strengthened upon viral infection; this triggers translocation of IFI16 to the cytoplasm and inflammasome activation^113^. In contrast, DNA viruses produce specific proteins that enable escape from IFI16-mediated immune responses. The HSV-1 viral E3 ubiquitin ligase ICP0 suppresses IFI16 by mediating its proteasomal degradation^114^. In addition, it has been reported that KSHV lytic protein(s) potentially degrade IFI16 to maintain latency^115^. Finally, HCMV possesses proteins that interfere with IFI16; Vps4 and TGN46 induce trafficking of IFI16 to multivesicular bodies, whereas pUL83 interacts with the PYRIN domain, which interferes with DNA sensing or inhibits expression of interferon-inducible genes^116–118^.

To date, few studies have been conducted on viral proteins that interfere with the recognition and signaling mechanisms of IFI16. However, as mentioned above, recent papers suggest the existence of a relationship between IFI16 and cGAS-cGAMP signaling^112,119^. A study in keratinocytes showed that IFI16 is required for STING activation by cGAMP and that IFI16 interacts with STING to promote its phosphorylation^112^, and there is another report that IFI16 interacts with the cGAS–STING pathway in macrophages^119^. These findings indicate that IFI16 is more closely related to DNA virus recognition and viral defense mechanisms than once thought. For this reason, it is expected that new virus proteins that interfere with the recognition and signaling mechanisms of IFI16 will be reported in the near future.

## AIM2 and viral evasion

AIM2, a member of the PYRIN protein family, consists of two domains: the PYRIN domain at the N-terminus and HIN200 domain at the C-terminus^81^. The HIN200 domain is responsible for DNA binding, whereas the PYRIN domain interacts with the PYRIN domain of ASC to activate caspase-1^74,81^. AIM2 has affinity for viral DNA derived from murine cytomegalovirus (MCMV) and Vaccinia virus; activation of AIM2 leads to secretion of IL-1β of IL-18 and mediates inflammation in response to viral infection^18,81^. As regulatory molecules, nuclear factor E2-related factor-2 (Nrf2) and pyruvate kinase isozyme M2 (PKM2) act as positive regulators of AIM2 inflammasome activation^120,121^. In contrast, high-mobility group box 1 (HMGB1) and DNA complexes induce autophagy to reduce activation of the AIM2 inflammasome^122^. TRIM11 mediates autopolyubiquitination and negatively regulates the AIM2 inflammasome by recruiting p62 and triggering selective autophagy^123^.

In contrast, pUL83 of HCMV binds to AIM2 and disrupts AIM2-mediated inflammasome activation. Upon HCMV infection, pUL83 interacts with AIM2 in macrophages, thereby inhibiting activated inflammasome components^124^. Recent reports show that VP22 of HSV-1 negatively regulates AIM2 inflammasome formation and IL-1β secretion: VP22 interacts with the HIN200 domain, but not with the PYRIN domain, to inhibit oligomerization of AIM2. Consequently, VP22-mediated inactivation of the inflammasome promotes virus replication in vivo^125^.

Taken together, these studies indicate that the AIM2 inflammasome serves as a key element in innate immunity against DNA viruses and that several viral proteins specifically inhibit AIM2 activation. Because the AIM2 inflammasome is also involved in the sensing of other DNA viruses, it is expected that further research will identify even more viral proteins that interact with AIM2. Furthermore, inflammasome activation not only constitutes a barrier to DNA viral infection but also is injurious to the host. In this respect, further studies are needed to determine the mechanisms that modulate the activity of the AIM2 inflammasome to enhance our understanding of host responses to DNA viral infection.

## Conclusions

Intracellular sensing of viral RNA or DNA by PRRs is indispensable for host cells to mount an antiviral innate immune response to inhibit replication and spread of invading viruses and prime an effective adaptive immune response^18,73^. This review summarizes our current knowledge of the key intracellular sensors and how they are modulated by various molecules to mediate IFN responses and the maintenance of immune homeostasis. Moreover, we discuss viral proteins that interact with these host cytosolic sensing molecules and facilitate evasion of host defenses.

Over the past decade, our understanding of intracellular sensor-mediated antiviral responses has expanded, and we know much more about the molecular mechanisms by which they are regulated via host and viral factors. This knowledge not only allows us to understand viral pathogenesis but also reveals how intracellular sensors are activated and regulated. Extensive knowledge of these mechanisms will allow for research and development of novel anti-inflammatory agents, immunostimulatory agents, new vaccines, and antiviral agents that target cellular regulators or specific viral proteins. Regardless, further work is needed to identify other cytosolic sensors (such as novel sensors of nucleic acids), other positive or negative regulatory molecules and related pathways, and novel escape mechanisms utilized by new viruses or variants.
